# Transcriptome Analysis of the Hierarchical Response of Histone Deacetylase Proteins That Respond in an Antagonistic Manner to Salinity Stress

**DOI:** 10.3389/fpls.2019.01323

**Published:** 2019-10-18

**Authors:** Minoru Ueda, Akihiro Matsui, Shunsuke Watanabe, Makoto Kobayashi, Kazuki Saito, Maho Tanaka, Junko Ishida, Miyako Kusano, Mitsunori Seo, Motoaki Seki

**Affiliations:** ^1^Plant Genomic Network Research Team, RIKEN Center for Sustainable Resource Science, Yokohama, Japan; ^2^Core Research for Evolutional Science and Technology (CREST), Japan Science and Technology Agency (JST), Kawaguchi, Japan; ^3^Plant Epigenome Regulation Laboratory, RIKEN Cluster for Pioneering Research, Wako, Japan; ^4^Dormancy and Adaptation Research Unit, RIKEN Center for Sustainable Resource Science, Yokohama, Japan; ^5^Metabolomics Research Group, RIKEN Center for Sustainable Resource Science, Yokohama, Japan; ^6^Graduate School of Pharmaceutical Sciences, Chiba University, Chiba, Japan; ^7^Graduate School of Life and Environmental Sciences, University of Tsukuba, Tsukuba, Japan; ^8^Kihara Institute for Biological Research, Yokohama City University, Yokohama, Japan

**Keywords:** high salinity stress, epigenetics, histone acetylation, histone deacetylases, stress response

## Abstract

Acetylation in histone and non-histone proteins is balanced by histone acetyltransferase and histone deacetylase (HDAC) enzymatic activity, an essential aspect of fine-tuning plant response to environmental stresses. HDACs in *Arabidopsis* are composed of three families (RPD3-like, SIRT, and HD-tuins). A previous study indicated that class I (HDA19) and class II (HDA5/14/15/18) RPD3-like family HDACs control positive and negative responses to salinity stress, respectively. Furthermore, quintuple *hda5/14/15/18/19* mutants (*quint*) exhibit salinity stress tolerance, suggesting that *hda19* suppresses the sensitivity to salinity stress present in quadruple *hda5/14/15/18* mutants (*quad*). In the present study, transcriptome analysis of the *quint* mutant was conducted to elucidate the hierarchical control of salinity stress response operated by RPD3-like family HDACs (HDA5/14/15/18/19). The analysis identified 4,832 salt-responsive genes in wild-type (Col-0), *hda19-3*, *quad*, and *quint* plants and revealed that 56.7% of the salt-responsive genes exhibited a similar expression pattern in both the *hda19-3* and *quint* plants. These results indicate that deficiency in HDA19 has a bigger impact on salinity stress response than in class II HDACs. Furthermore, the expression pattern of genes encoding enzymes that metabolize phytohormones raises the possibility that a drastic change in the homeostasis of phytohormones, such as abscisic acid, brassinosteroid, and gibberellin, may contribute to increasing stress tolerance in *hda19-3* and *quint* plants. Among these phytohormones, abscisic acid accumulation actually increased in *hda19-3* and *quint* plants, and decreased in *quad*, compared with wild-type plants. Importantly, 7.8% of the salt-responsive genes in *quint* plants exhibited a similar expression pattern in *quad* plants, suggesting that some gene sets are regulated in an HDA5/14/15/18-dependent manner. The transcriptome analysis conducted in the present study revealed the hierarchical and independent regulation of salt stress response that is mediated through HDA19 and class II HDACs.

## Introduction

Recent studies have demonstrated that a diverse type of histone modifications, such as acetylation, methylation, and phosphorylation, play a pivotal role in orchestrating plant response to environmental stresses (Kim et al., 2015). Acetylation levels are balanced by histone acetyltransferases (HATs) and histone deacetylases (HDACs). These enzymes have the ability to write or erase an acetylation mark, respectively. In general, the mark is positively correlated with mRNA expression. Notably, the mechanism by which histone acetylation governs plant response to environmental stresses through HDACs has been elucidated (Asensi-Fabado et al., 2016; Luo et al., 2017; Ueda et al., 2018b).

Three families of HDAC proteins (Reduced potassium dependency 3 (RPD3)-like, Silent Information Regulator 2 (SIRT), and HD-tuins) have been recognized in plants. The *Arabidopsis thaliana* genome encodes 18 genes that are members of the three HDAC families: 12 RPD3-like family proteins; 2 SIRT family proteins; and 4 HD-tuin family proteins. The RPD3-like family is further divided into three classes (I, II, and IV) based on their homology to yeast HDACs (Hollender and Liu, 2008). The RPD3-like family HDACs are pharmacological targets because their inhibition has potential application as a cancer treatment (Bolden et al., 2006; Seto and Yoshida, 2014). The potential value of their inhibition not only applies to cancer therapy but may also be applicable for increasing environmental stress tolerance in plants. *Arabidopsis* and cassava plants treated with HDAC inhibitors exhibit an increase in tolerance to salinity stress (Sako et al., 2016; Patanun et al., 2017; Nguyen et al., 2018). Pharmacological analysis indicated that inhibition of class I RPD3-like HDACs is essential for increasing salinity tolerance based on the evaluation of the survival of plants treated with class-selective inhibitors and subjected to salinity stress conditions (Ueda et al., 2017).

Consistent with the pharmacological studies, *Arabidopsis* plants deficient in class I RPD3-like HDACs (HDA6, HDA9, and HDA19) exhibit increased tolerance to environmental stresses, including different accessions of Col-0. A previous study reported that *hda19* plants (*hda19-3* and *hda19-5*) in the Col-0 background exhibit tolerance to salinity, drought, and heat stress (Ueda et al., 2017; Ueda et al., 2018a). In *hda19-3* plants, positive regulators, such as *NAC019* and *ABI5* in the ABA signaling pathway, that contribute to enhanced environmental stress tolerance are strongly upregulated (Ueda et al., 2017). HDA6 and HDA9 negatively regulate the expression of drought stress tolerance-related genes (class I RPD3-like HDAC) (Zheng et al., 2016; Kim et al., 2017). HDA9 is also involved in improving salt stress tolerance (Zheng et al., 2016). Collectively, the data indicate that inhibition of class I RPD3-like HDACs plays an essential role in improving environmental stress tolerance and that they function as a negative regulator of increased tolerance to environmental stresses, with the exception of the hypersensitive phenotype of the *hda6* mutant in response to salt stress (Chen et al., 2010). In contrast to the inhibition of class I RPD3-like HDACs, the multiple inhibition of class II RPD3-like HDACs (*quadruple hda5/14/15/18* mutant: *quad*) decreases salinity stress tolerance (Ueda et al., 2017). Thus, these previous studies imply that HDACs participate in an antagonistic manner in response to salinity stress.

Although the different classes of HDACs regulate salinity stress responses in a contrasting manner, the epistasis of *hda19-6* in *quad* has been observed. When a mutation of HDA19 is introduced in *quad*, the mutation converts the salt-sensitive phenotype to a salt-tolerant phenotype (Ueda et al., 2017). The details of which quintuple *hda5/14/15/18/19* mutant (*quint*) acquired the tolerance to salinity stress, however, remain unknown. In the present study, a genome-wide transcriptional profiling of *quint* was conducted to reveal the molecular mechanism underlying the epistasis of HDA19 against HDA5/14/15/18. The transcriptional profiling identified 4,823 salt-responsive genes in samples of whole seedlings that exhibited significant changes in expression in Col-0, *hda19-3*, *quad*, and *quint* plants. Further analysis indicated that 56.7% of the salt-responsive genes exhibited a similar expression pattern in both *hda19-3* and *quint* plants. Notably, genes for abscisic acid (ABA) biosynthesis were significantly upregulated in *quint* and *hda19-3* plants growing under non-stress and/or salinity stress conditions. In accordance with the transcriptional profiling, their ABA levels increased under salinity stress conditions. This was in addition to the upregulation of genes for transcriptional regulators, such as ABI5, reported in a previous study, suggesting that earlier expression of ABA signaling pathway genes contributes to increasing salinity tolerance in *hda19* and *quint* plants. The results of the transcriptional profiling also indicate that an alteration of phytohormone homeostasis, in particular ABA, brassinosteroid (BR), and gibberellin (GA), occurs in *hda19* and *quint* plants.

The effects of salinity stress on plant growth could be divided into two types. Initially, osmotic stress has an impact on plant water uptake after onset of the salt stress, and, subsequently, specific ion toxicities cause to reduce cellular metabolism (Munns et al., 2006). Here, we mainly evaluate the effect of osmotic stress on plant growth because 5-day-old plants are subjected to higher salinity stress conditions (125 mM NaCl). The results indicate that 7.8% of the salt-responsive genes exhibit a similar expression pattern in *quint* and *quad* plants. Among them, the expression of *NAC016*, which is involved in leaf senescence (Kim et al., 2013), is strongly induced in *quad* plants and is considered to play a major role in producing the salt stress-sensitive phenotype. The upregulation of *NAC016*, however, was detected in *quint* mutant plants that exhibit similar tolerance to salinity stress to *hda19* plants, suggesting the presence of an HDA5/14/15/18 regulatory network independent from HDA19. The transcriptional profile conducted in the present study identified a set of genes that were regulated counteractively in *quad* and *quint* plants, which resulted in the enrichment of genes encoding enzymes involved in stress sensitivity (e.g., *IPT7* and *ISOPENTENYL TRANSFERASE 7*). The mode of action of HDA19 and HDA5/14/15/18 in salinity stress response and where crosstalk occurs between them during salinity stress response are discussed.

## Materials and Methods

### Plant Material and Growth Conditions

*Arabidopsis thaliana* (L.) Heynh. (Columbia: Col-0), *hda19-3*, *hda5*/*14*/*15*/*18* (*quad*) mutant, and *hda5*/*14*/*15*/*18*/*19* (*quint*) mutant plants were used in this study. *quint* has the *hda19-6* allele. Details pertaining to the wild-type and mutant plants have been previously reported (Ueda et al., 2017). The T-DNA insertional mutants for the generation of the listed mutants were obtained from ABRC (Samson et al., 2002; Alonso et al., 2003). After surface sterilization with sodium hypochlorite, followed by two rinses with distilled water, seeds were floated on 1 ml liquid media (half-strength MS medium with 0.5% MES and 0.1% agar, pH 5.7) for 48 h at 4°C in 24-well tissue culture plates (TPP; Trasadingen, Switzerland). After germination, the seedlings in the 24-well tissue culture plates were placed on a cultivation rack at 22°C under a long-day photoperiod (16h:8h light/dark cycle) at 70–90 μE m^− 2^ s^− 1^. When plants were 5 days old (counted after seeds had germinated), the treated plants in each well were administered 25 μl 5 M NaCl (125 mM NaCl per well), while the controls received no salt treatment. Extraction of ABA, proline, and RNA from salt-treated and non-treated (control) plants was conducted for the following analysis at 2 h after the salt treatment was administered. In the evaluation of salinity tolerance in the *hda19-3*, *quad*, and *quint* mutants, 100 mM NaCl treatments were applied to 5-day-old plants (counted after seeds had germinated) in liquid culture and the percentage survival determined 4 days later (three biological replicates consisted of 10 plants; means ± SD). Plants with green true leaves were counted as a survival. Significant differences between the survival values of the experimental plants, relative to untreated or NaCl-treated wild-type plants, were determined using Student’s *t* test (*P < 0.05 and **P < 0.01).

### Microarray Analysis

Total RNAs were extracted from 5-day-old plants using an RNeasy Plant Mini Kit (Qiagen, Valencia, CA). All RNAs were further purified by incubation with RNase-free DNase I (Qiagen), according to the manufacturer’s instructions. RNAs were reverse transcribed into cDNAs using 400 ng of total RNA. cDNA was labeled with a single color (Cy3) using a Quick Amp labeling kit (Agilent Technologies, Palo Alto, CA, USA) and hybridized to an *Arabidopsis* custom microarray (GEO array platform: GPL19830, Agilent Technologies) (Nguyen et al., 2015). Arrays were scanned with a microarray scanner (G2505B, Agilent Technologies). The resulting microarray data were deposited in and are available on the GEO website (GEO ID: GSE121225). The R program ver. 3.2.3 was used for the analysis of the microarray data. The fluorescence intensities of the microarray probes were normalized by quantile normalization using the limma package (Smyth, 2004). Genes with a significant change in expression were selected using the following criteria: an expression log_2 ratio >0.5 and a controlled *p* value (false discovery rate, FDR; Benjamini and Hochberg, 1995) from a *t* test analysis <0.05. For construction of the heat map, a *Z* score was computed for each of the selected genes using gplots. For constructing the hierarchical cluster, pairwise distances between the expression data of significant expression changed genes were calculated using the “Euclidean” method and hierarchical clustering on this distance matrix was constructed using the “Ward” method. The resultant was divided into 10 groups by using the “cutree” function in R.

### RT-qPCR Analysis

First-strand cDNA was synthesized from 250 ng total RNA with random primers. ReverTra Ace (TOYOBO) was used for the reverse transcription reaction according to the manufacturer’s instructions. Transcript levels were assayed using THUNDERBIRD SYBR qPCR Mix (TOYOBO) and a StepOnePlus Real-Time PCR System (Applied Biosystems) according to the manufacturer’s protocols. Gene-specific primers were designed using the PrimerQuest tool (http://sg.idtdna.com/primerquest/Home/Index). Melting curve analyses were conducted to validate the specificity of the PCR amplification. At least three biological replicates were used in each reverse transcription–quantitative PCR (RT-qPCR) assay. *MON1* was used as a reference gene to normalize data because this gene has been demonstrated to be one of the best reference genes for *Arabidopsis* (Remans et al., 2008). The microarray data also support the use of this reference gene under the experimental conditions used in this study. The RT-qPCR scores and relevant primers are listed in **Supplementary Tables S1** and **S2**, respectively.

### ABA Measurement

Endogenous ABA was extracted with 80% (*v*/*v*) acetonitrile containing 1% (*v*/*v*) acetic acid from whole wild-type and mutant seedlings after freeze drying. Hormone contents were determined using a UPLC-MS/MS system consisting of a quadrupole/time-of-flight tandem mass spectrometer (Triple TOF 5600, SCIEX, Concord, Canada) and a Nexera UPLC system (Shimadzu Corp., Kyoto, Japan) as described previously (Kanno et al., 2016).

### Proline Measurement

Quantification of proline content was conducted as described in Kusano et al. (2007). Briefly, each frozen sample was extracted with a concentration of 25 mg fresh weight (FW) of whole seedling per milliliter of extraction medium (methanol/chloroform/water, 3:1:1 *v*/*v*/*v*) containing 10 stable isotope reference compounds. An equivalent of the 27.8 µg FW of the derivatized sample was injected to Leco Pegasus HT gas chromatography–time-of-flight mass spectrometry (LECO, St. Joseph, MI, USA). Data processing was performed using ChromaTOF version 4.72.0.0 (LECO, St. Joseph, MI, USA). A calibration curve was generated by analyzing the derivatized proline at concentrations of 0.1, 0.5, 1.0, 3.0, 5.0, and 10 ng/µl of injection buffer, respectively. We applied specific ions at *m/z* 142 and 216 to quantify proline content. For normalization, 15 ng/µl of [^13^C_5_]-proline (Cambridge Isotope Laboratories, Andover, MA, USA) was used. We chose *m/z* 146 and 220 for it.

### Statistical and GO Enrichment Analysis

**Figure 1:**
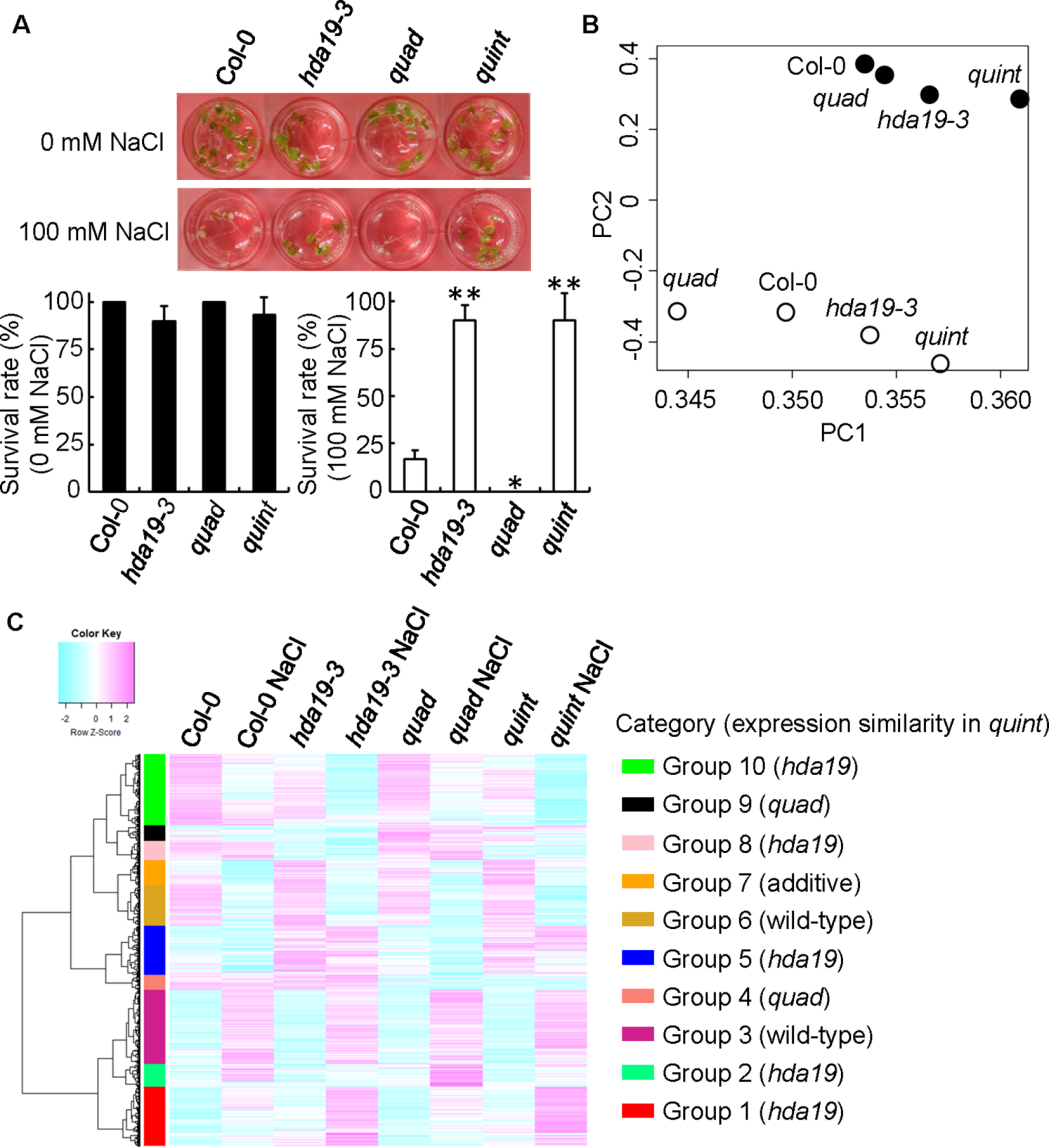
Microarray analysis of genome-wide transcription in Col-0, *hda19-3*, *quad*, and *quint* plants under non-stressed and salinity-stressed growth conditions. **(A)** The survival rate (in percent) of each plant was evaluated 4 days after treatment with 100 mM NaCl or without NaCl (means ± SD; *n* = 3, where each biological replicate was a collection of 10 plants). *P* values were calculated using Student’s *t* test (*P < 0.05 and **P < 0.01). **(B)** Principal component analysis (PCA) based on whole-genome transcriptome analysis. *Filled* and *open circles* reflect the expression profiles under normal and salinity growth conditions, respectively. **(C)** Hierarchical cluster analysis of salt-responsive genes. The genome-wide mRNA profiles determined by microarray analysis were obtained from 5-day-old plants treated with or without 125 mM NaCl for 2 h. Transcript data were generated from three biological replicates. The heat map represents the *Z* score, with *bars* showing values from −2 to 2. *Red* represents upregulated genes, while *blue* represents downregulated genes. Genes with a significant change in expression were selected using the following criteria: an expression log_2 ratio >0.5 and a controlled *p* value (FDR; Benjamini and Hochberg, 1995) from a *t* test analysis <0.05.

Changes in gene expression and ABA and proline accumulations were statistically analyzed with one-way ANOVA from data obtained from three or four biological replicates. P < 0.05 was considered as significant. Gene ontology (GO) analyses of upregulated genes in *hda19* (Group 1 in **Figure 1C**) and *quad* (Group 2 in **Figure 1C**) based on results obtained from the above microarray analysis were carried out using PANTHER (Mi et al., 2013).

### Accession Numbers

Sequence data from this article can be found in the *Arabidopsis* Genome Initiative or GenBank/EMBL databases under the following accession numbers: *ABA2*; AT1G52340, *ABI5*; AT2G36270, *GA 2ox7*; AT1G50960, *GA 20ox1*; AT4G25420, *IPT7*; AT3G23630, *MON1*; AT2G28390, *NAC016*; AT1G34180, *NCED4*; AT4G19170, *UGT73C5*; AT2G36800.

## Results

### Global Transcriptome Analysis in Response to Salinity Stress

Under salinity stress conditions, the *hda19-3* and *quad* mutant plants showed a higher and a lower survival rate than wild-type (Col-0) plants, respectively. Similar to the *hda19-3* mutant plants, the *quint* plants exhibited a comparable survival rate (**Figure 1A**). To understand responses to salt stress and elucidate the details of the mechanism underlying increased tolerance to salinity stress in *quint*, transcriptional profiling using a genome-wide microarray was conducted for *quint* (*hda19-6*, an allele of *hda19*), *hda19-3*, and *quad* mutant plants, as well as wild-type plants, growing under normal and salinity stress conditions. The analysis identified 4,823 salt-responsive genes in whole seedling samples that exhibited significant expression changes in at least one of the genotypes (one-way ANOVA with Benjamini–Hochberg correction [FDR] < 0.05; **Figures 1B, C**). A principal component analysis (PCA) of gene expression was conducted to understand the global expression patterns of the 4,823 salt-responsive genes in the mutant and wild-type plants under salt stress and non-stressed conditions. The results indicated that the *quad* mutant does not cluster with the wild type anymore after salt stress (open circles in **Figure 1B**). Furthermore, the clustering between *hda19-3* and *quint* is very similar between the control (filled circles in **Figure 1B**) and salt (open circles in **Figure 1B**) conditions. These results indicate that the global expression pattern in the *quint* mutant in response to salinity stress was similar to the response of the *hda19-3* mutant.

The heat map illustrates that there are significant differences in the transcriptional accumulation of the 4,823 salt-responsive genes under non-stress and salinity stress conditions in wild-type plants and each mutant genotype. A hierarchical cluster analysis revealed that differentially expressed genes in each condition can be divided into ten groups (Group 1: 729, Group 2: 281, Group 3: 903, Group 4: 183, Group 5: 607, Group 6: 498, Group 7: 304, Group 8: 239, Group 9: 193, and Group 10: 881 genes; **Figure 1C** and **Supplementary Tables S3–S12**). The lack of HDAC (*HDA19* or *HDA5/14/15/18*) expression had no significant effect on the expression of the collective 1,401 genes in Groups 3 and 6. The remainder of the 4,823 salt-responsive genes was affected under non-stress and/or salt stress conditions by the genetic defects. As indicated by the PCA, *hda19* is clearly pleiotropic over the *quad*. A total of 56.7% of the salt-responsive genes (Groups 1, 2, 5, 8, and 10) exhibited a similar expression pattern in both *hda19-3* and *quint* plants. In contrast, only 7.8% of the salt-responsive genes (Groups 4 and 9) exhibited a similar expression pattern in *quad* and *quint* plants. Notably, Group 7 enriched genes were expressed additively in *quint* plants relative to *hda19-3* and *quad* plants, suggesting that *HDA19* and *HDA5/14/15/18* redundantly act on mRNA expression. Importantly, however, HDA19 and HDA5/14/15/18 mainly regulate a set of genes in response to salinity stress in an independent manner.

### Upregulation of ABA Signaling Pathway-Related and ABA Metabolism-Related Genes That Are Independently Regulated by HDA19 in *hda19-3* and *quint* Mutants

**Figure 2:**
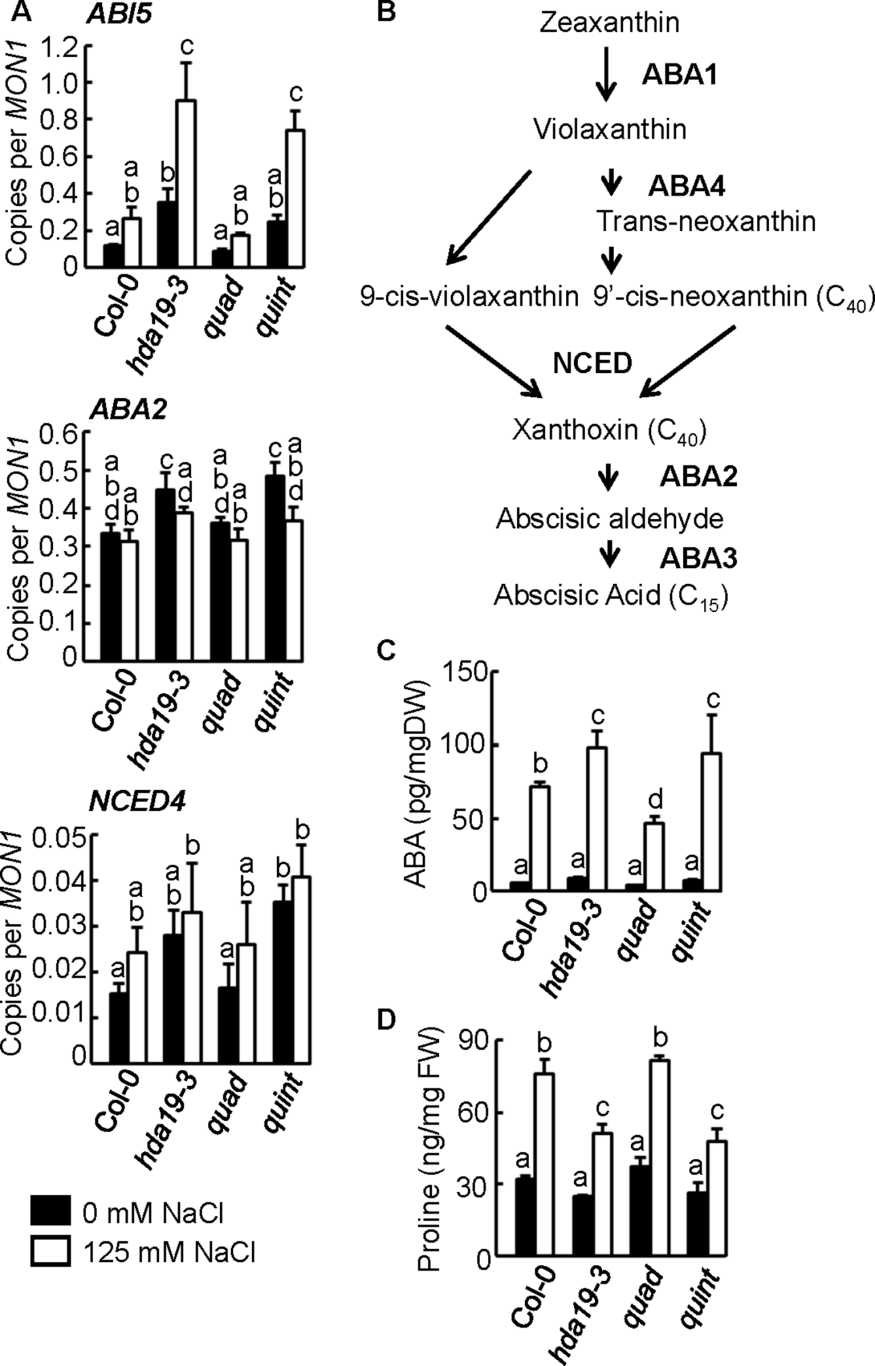
Upregulation of *ABI5* and ABA biosynthesis genes, such as *ABA2* and *NCED4*, and increased ABA and decreased proline accumulation in *hda19-3* and *quint* mutants. **(A)** Expression profiles of *ABI5*, *ABA2*, and *NCED4* genes using reverse transcription–quantitative PCR (RT-qPCR) analysis. Tissue samples of 5-day-old seedlings of wild-type (Col-0), *hda19-3*, *quad*, and *quint* plants growing under non-stressed (*black columns*) or salinity stress (125 mM NaCl, *white columns*) conditions for 2 h were collected and analyzed by RT-qPCR. Multiple comparisons of RT-qPCR scores were performed with one-way ANOVA. *P* < 0.05 was considered as significant. *MON1* (AT2G28390) was used as the reference gene. Three independent biological replicates of each line were analyzed for each condition. **(B)** Simplified schematic representation of the ABA biosynthesis pathway. **(C**, **D)** Measurement of ABA **(C)** and proline **(D)** accumulation in 5-day-old seedlings of wild-type (Col-0), *hda19-3*, *quad*, and *quint* plants growing under non-stressed (*black columns*) and salinity stress conditions (*white columns*). Multiple comparisons of ABA and proline contents from four and three replications, respectively, were performed with one-way ANOVA. *P* < 0.05 was considered as significant.

In general, histone deacetylases catalyze the deacetylation of histone tails, leading to DNA compaction tightening, inducing a transcriptional inactivation; hence, one would expect that mutation of HDACs has the opposite effect: transcriptional activation. GO enrichment analysis revealed that the majority of the genes in Group 1 (**Figure 1C**) containing upregulated genes under salinity stress conditions in *hda19* and *quint* compared with wild-type plants belongs to GO:0009611 (response to wounding), GO:0009414 (response to water deprivation), GO:0009651 (response to salt stress), and GO:0009737 (response to abscisic acid; **Supplementary Table S13**). Consistent with our previous study, transcription factors such as ABI5, which are considered to be a major positive regulator in the ABA signaling pathway, were strongly upregulated in both *hda19-3* and *quint* plants subjected to salinity stress conditions (Group 1 in **Figure 1C** and **Figure 2A**; **Supplementary Table S1**). In addition to the transcriptional factors involved in ABA signaling, the transcriptional profiling analysis revealed that the ABA biosynthesis genes *ABA2* and *NCED4* were also upregulated in *quint* and/or *hda19-3* plants under non-stress and/or stress conditions (**Figures 2A, B** and **Supplementary Table S14**). Their upregulation was also confirmed by RT-qPCR (**Figure 2A** and **Supplementary Table S1**). In contrast, the upregulation of *ABA2* and *NCED4* was not observed in the transcriptional profiling analysis of *quad* plants (**Figure 2A** and **Supplementary Table S1**). Consistent with the transcriptome analysis, relative to wild-type plants, the ABA levels increased in *hda19-3* and *quint* plants. Decreased accumulation of ABA in *quad* under salinity stress conditions was detected, although our transcriptome analysis could not find any signs of the decline to the best of our knowledge (**Figure 2C** and **Supplementary Table S1**). Thus, it appears that HDA19 exerts its anti-stress effect through its regulation of ABA signaling, which operates in a class II HDAC-independent manner.

ABI5 functions at the core of ABA signaling, and the induction of ABI5 expression causes growth arrest and the induction of some of the LATE EMBRYOGENESIS ABUNDANT (LEA) proteins, such as RAB18, COR6.6, COR15A, EM1, and EM6, that prevent protein aggregation resulting from water loss at all stages of plant growth (Skubacz et al., 2016). Only the induction of *COR6.6* was almost significant (*p* < 0.058) under normal growth conditions and was clearly significant under salinity stress conditions (*p* < 0.016). Nonetheless, *EM6* was one of the direct targets (Finkelstein and Lynch, 2000), while the expression of the remaining four *LEA* genes did not increase significantly (**Supplementary Table S15**). These data suggest that an atypical activation of ABA signaling, coordinated by an absence of *HDA19* expression, may occur in *hda19* and *quint* plants.

To characterize the phenotype of mutants, we measured the accumulation of proline, known to act as a compatible osmolyte to counteract salinity stress (Szabados and Savoure, 2010). Salinity stress induced proline accumulation in all lines, and, under salinity stress conditions, there were significant declines in proline accumulation in *hda19-3* and *quint* plants compared with wild-type and *quad* plants (**Figure 2D**).

### Altered Expression of Genes Encoding Enzymes Related to Phytohormone Biosynthesis or Catabolism

**Figure 3:**
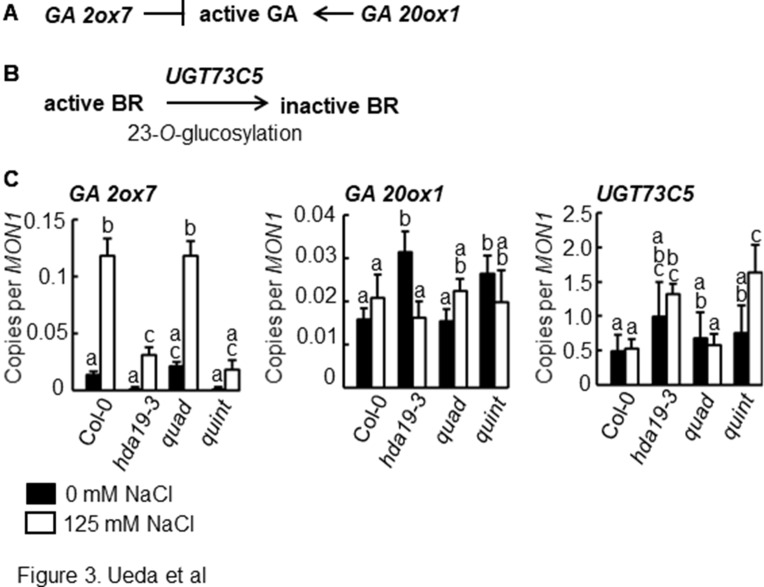
Altered expression patterns of genes for GA- and BR-catabolizing enzymes in *hda19* and *quint* mutants. **(A)** GA2ox7 and GA20ox1 catalyze the degradation and synthesis of bioactive GA, respectively. **(B)** UGT73C5 catalyzes the 23-*O*-glucosylation of BRs, resulting in the biosynthesis of inactive BRs. **(C)** RT-qPCR analysis of *GA2ox7*, *GA20ox1*, and *UGT73C5* gene expressions. Experimental conditions and statistical analysis of RT-qPCR data were the same as shown in **Figure 2A**.

Phytohormones play an important role in fine-tuning abiotic stress response in plants (Qin et al., 2011). GA is believed to counteract the effect of ABA. Thus, fine-tuning the ABA/GA ratio is essential to regulate both plant development and plant response to environmental stress (Vanstraelen and Benkova, 2012). Microarray data indicated that the expression of genes involved in GA biosynthesis was altered in *hda19-3* and *quint* plants. *GA2ox7*, a GA catabolic gene that degrades bioactive GA under salinity stress conditions (Magome et al., 2008; Colebrook et al., 2014), was significantly suppressed in *hda19-3* and *quint* plants under salinity stress conditions (**Figures 3A, C** and **Supplementary Table S1**). The expression of *GA20ox1*, which encodes a key oxidase enzyme in the biosynthesis of GA (De Vleesschauwer et al., 2012), was significantly upregulated in *hda19-3* and *quint* plants relative to wild-type plants under non-stress conditions (**Figures 3A, C** and **Supplementary Table S1**).

BR/ABA antagonism is known to play a role in balancing growth and stress response in plants (Clouse, 2016). BR homeostasis appeared to be altered in *hda19-3* and *quint* plants. UGT73C5, which catalyzes BL-23-*O*-glucosylation and a subsequent reduction in BR activity (Poppenberger et al., 2005), was significantly upregulated under salinity stress conditions (**Figures 3B, C** and **Supplementary Table S1**). These data indicate that the homeostasis of phytohormones, in particular ABA and ABA-antagonistic phytohormones such as GA and BR, may be altered in *hda19-3* and *quint* plants.

### Presence of an HDA19-Independent Pathway and a Pathway Counteractive to HDA19 Coordinated by Class II HDACs

**Figure 4:**
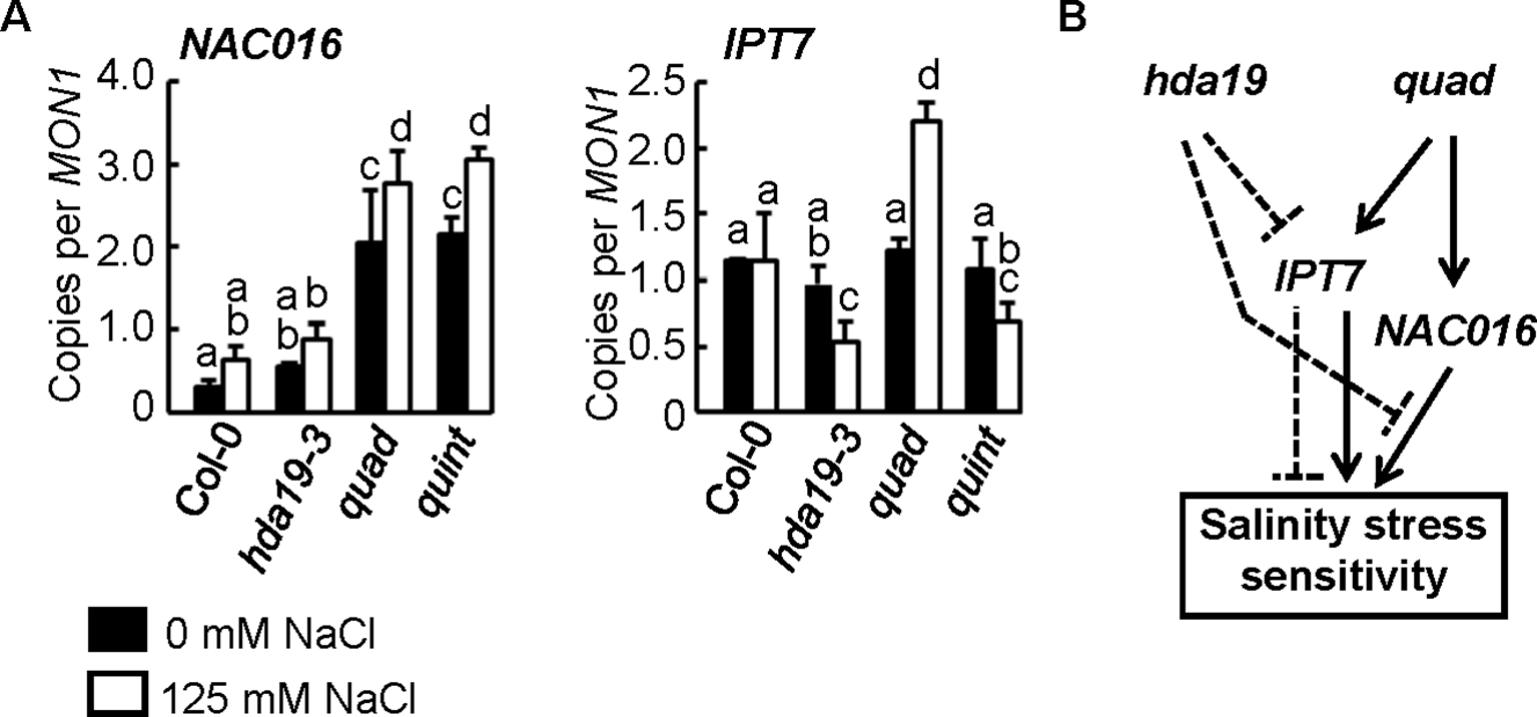
HDA5/14/15/18-dependent regulation of *NAC016* and counteractive regulation of *IPT7* between *hda19* and* quad*. **(A)** Reverse transcription–quantitative PCR (RT-qPCR) analysis of the expressions of *NAC016* and *IPT7* genes. Experimental conditions and statistical analysis of RT-qPCR data were the same as shown in **Figure 2A**. **(B)** A simplified model for the counteractive and hierarchical control of the expressions of *NAC016* and *IPT7* genes mediated through HDA19 and HDA5/14/15/18 deficiencies.

We have not detected any explainable genes from Group 2 (**Figure 1C**) containing upregulated genes under salinity stress conditions in *quad* compared with wild-type plants by the GO enrichment test (**Supplementary Table S16**). However, *NAC016*, whose expression is involved in senescence (Kim et al., 2013), was strongly induced in *quad* plants (Ueda et al., 2017). *Arabidopsis* plants overexpressing *NAC016* (*ANAC016-OX*) rapidly turn white when subjected to either salt or oxidative stress (Kim et al., 2013). Consistent with a previous study (Ueda et al., 2017), the *quad* plants in the current study were sensitive to salinity stress (**Figure 1A**). Unexpectedly, the significant induction of *NAC016* was retained in *quint* plants, although *quint* plants exhibited an elevated level of salinity tolerance that is similar to *hda19-3* plants (**Figure 4A**, Group 9 in **Figure 1C**, **Supplementary Table S1**). This suggests that there is an independent mechanism regulating *NAC016* expression in HDA5/14/15/18 (*quad*) plants that does not involve HDA19.

Transcriptional analysis detected a set of genes that were regulated counteractively in *hda19* alleles (*hda19-3* and *quint*) and *quad* (Groups 2 and 8 in **Figure 1C**; **Supplementary Tables S4** and **S10**) plants, which could account for the discrepancies in the upregulation of *NAC016* and the salt-tolerant phenotype in *quint*. Deletions of *HDA5/14/15/18* activate the expression of the cytokinin (CK) biosynthetic gene, *ISOPENTENYL TRANSFERASE7* (*IPT7*), resulting in increased levels of CK (Yanai et al., 2005) (**Supplementary Table S4**). Overproduction of endogenous cytokinin and decreased levels of CK have been reported to decrease and increase salt tolerance, respectively (Nishiyama et al., 2012; Wang et al., 2015). Downregulation of *IPT7* was observed in the current study by the introduction of the *HDA19* mutation into *quad* plants under salinity stress conditions (**Figure 4A** and **Supplementary Table S1**). This counteractive expression of *IPT7* may reflect the opposite salt sensitivity and salt-tolerant phenotypes observed in *quad* and *quint* plants, respectively (**Figure 4B**). 

## Discussion

ABA signaling is one of the major phytohormonal pathways responsible for increasing tolerance to a variety of environmental stresses, including salinity and drought (Larosa et al., 1987; Mantyla et al., 1995; Bari and Jones, 2009; Vishwakarma et al., 2017). The current study indicated that ABA signaling is strongly induced in *hda19* mutant plants. There is no clear morphological difference between *quad* and wild-type plants. On the other hand, *hda19-3* and *quint* showed lower germination rates under non-stress conditions, although statistical significance was not detected (**Figure 1A**). A wide range of developmental abnormalities such as growth inhibition was observed in *hda19-3* (Hollender and Liu, 2008). Some of them might be explainable by the enhanced acclimation of ABA. The transcriptome analyses revealed that an HDA19 defect primarily activates the ABA signaling pathway, which is independently regulated from class II HDACs. ABA signaling plays a pivotal role in the growth reduction of plants during the early phase of plant response to salinity stress (Ismail et al., 2014). In contrast, deficiencies in class II HDAC genes appear to result in a malfunction in the growth reduction because expansin 12 (a cell wall-loosening protein; **Supplementary Table S4**) (Lee et al., 2001) and TRH1 (K^+^ transporter for root hair elongation; **Supplementary Table S4**) (Desbrosses et al., 2003) are activated in response to salinity stress conditions in *quad* plants as in the case of *IPT7* (**Figure 4**). In *hda19-3* and *quint* plants, the upregulation of these genes could not be detected (**Supplementary Tables S4** and **S10**). The HDA19 defect, in which HDA19 is not produced, may induce an earlier expression of genes involved in the ABA signaling pathway, including ABA metabolism and other transcriptional factors.

BR homeostasis appeared to be altered in *hda19* and *quint* plants. UGT73C5, which catalyzes BL-23-*O*-glucosylation leading to reduced BR activity (Poppenberger et al., 2005), was significantly upregulated under salinity stress conditions (**Figure 3C**). Under the experimental conditions used in the current study, ABA signaling may be activated due to the reduced accumulation of active BR, which results in increased salinity stress tolerance. In support of this premise, *ABI5* expression has been reported to directly suppress BR signaling through the BRASSINAZOLE RESISTANT 1 (BZR1) transcription factor (Yang et al., 2016). The upregulation of *ABI5* expression under salinity stress conditions may indicate the reduced accumulation of active BRs in *haa19* and *quint* plants. A previous study also indicated that BR signaling is involved in the suppression of GA biosynthetic genes, such as *GA20ox* and *GA3ox3*, and the induction of a GA inactivation gene, such as *GA2ox*, which leads to the suppression of GA signaling (De Vleesschauwer et al., 2012). In the current study, the transcriptome analysis indicates that GA signaling may be activated in *hda19* and *quint* plants because suppression of *GA2ox7* and the induction of *GA20ox1* were detected (**Figure 3C**). Considering the above data, a reduced accumulation of active BRs may trigger a change in the expression pattern of genes encoding phytohormone-catabolizing enzymes.

In contrast to ABA metabolism, a defect in GA metabolism generally enhances tolerance to abiotic stresses, such as salinity and drought stress (Achard et al., 2006; Colebrook et al., 2014). The results of the present study indicate that GA metabolism is activated in *hda19* and *quint* plants based on the transcriptome analysis (**Figure 3C**). These plants, however, exhibit tolerance to salinity stress. Furthermore, *hda19* plants also exhibit tolerance to drought stress (Ueda et al., 2018a). If the hormone network is altered in *hda19-3* or *quint* plants, the altered hormone network (coincidental activation of antagonistic hormone signaling of ABA and GA) may allow plants to alleviate growth arrest induced by ABA signaling. Notably, *ga2ox7* mutants that accumulate active GA exhibit less growth retardation than Col wild-type plants of *Arabidopsis* subjected to high-salinity stress (Magome et al., 2008). ABA stimulates generally an increase in proline accumulation (Szabados and Savoure, 2010). ABA increased in *hda19* and *quint*; nonetheless, a lower proline accumulation was found in them than in wild-type plants and *quad* (**Figure 2D**). The unusual response in proline accumulation might be due to the alteration in phytohormone homeostasis under salinity stress conditions. Further studies are needed to reveal how epigenetic regulation mediated by HDA19 controls stress responses and crosstalk between phytohormones.

We confirmed the altered mRNA expressions of *ABI5*, *ABA2*, *NCED4*, *GA 2ox7*, *GA 20ox1*, *UGT73C5*, *NAC016*, and *IPT7* genes under normal and/or salinity growth conditions by microarray and RT-qPCR analysis. Among them, we found tissue- or organ-specific expressions, except for *ABA2* and *GA 2ox7* genes, by *Arabidopsis* eFP Browser (Winter et al., 2007) (**Supplementary Figure S1**). In the study, we have not revealed whether their alterations are due to the derepression of their tissue-specific expression or activation through the inhibition of HDAC activity. A detailed analysis of their expression is needed to understand how HDAC inhibition alters salinity stress response.

HDA19 mainly appears to mediate histone H3 acetylation (Zhou et al., 2005; Krogan et al., 2012). In class II HDACs, HDA5 participates in histone H3 acetylation (Luo et al., 2015). HDA15 participates in histone H3 and H4 acetylation (Liu et al., 2013; Gu et al., 2017). HDA14 is located in chloroplasts, and its localization to chromatin has not been documented (Alinsug et al., 2012). Synergistic upregulation of HDA19 and HDA5/14/15/18 deficiencies observed in *quint* plants may be due to the redundancy in histone or non-histone acetylation coordinated by both HDA19 and class II HDACs. As shown in **Figure 4A**, the expression of *NAC016* is perhaps independent of HDA19, as the majority of the responses described in the manuscript seem to be otherwise. In the case of HDA19- and HDA5/14/15/18-dependent regulation (HDA19- and HDA5/14/15/18-dependent expressions, categories 1, 2, 5, 8, and 10 and categories 4 and 9, respectively, in **Figure 1C**), levels in the acetylation of histones around target sites or proteins, including non-histones, may be independently coordinated by these HDACs. In actuality, multiple acetylations of non-histone proteins have been documented using HDAC inhibitors or mutants (Hao et al., 2016; Hartl et al., 2017). For example, it remains unclear whether the altered expression of genes for BR, GA, and other hormone pathways is due to the HDA19 action or a reflection of the effect on ABA signaling. Further analysis to determine whether HDACs directly regulate the gene expression of candidate signaling pathways through histone acetylation and/or are involved in the acetylation of key enzymes involved in hormonal crosstalk, as highlighted in the current study, will elucidate the detailed processes in stress responses in plants that are coordinated by HDA19 and HDA5/14/15/18.

## Author Contributions

MU and MSek designed the experiments. MU, AM, SW, MKo, MT, and JI conducted the experiments. MU, AM, SW, MKo, MKu, KS, and MSeo analyzed the data. MU, AM, SW, MKo, MKu, MSeo and MSek wrote the manuscript.

## Funding

This work was supported by grants to MU and MSek from the RIKEN and grants to MSek from Japan Science and Technology Agency (JST) [Core Research for Evolutionary Science and Technology (CREST, grant number JPMJCR13B4)], and KAKENHI on Innovative Areas (grant no. 16H01476, 18H04791, and 18H04705) of the Ministry of Education Culture, Sports and Technology of Japan.

## Conflict of Interest

The authors declare that the research was conducted in the absence of any commercial or financial relationships that could be construed as a potential conflict of interest.

## Supplementary Material

Figure S1 eFP diagrams of *ABI5*, *ABA2*, *NCED4*, *GA 2ox7*, *GA 20ox1*, *UGT73C5*, *NAC016*, and *IPT7* genes
