# Supplementary material for: Transcriptome Analysis of the Hierarchical Response of Histone Deacetylase Proteins That Respond in an Antagonistic Manner to Salinity Stress
Source: Front Plant Sci. 2019 Oct 18;10:1323. doi: 10.3389/fpls.2019.01323 (PMC6813852; doi:10.3389/fpls.2019.01323)
Supplement: Figure S1 — eFP diagrams of ABI5, ABA2, NCED4, GA 2ox7, GA 20ox1, UGT73C5, NAC016, and IPT7 genes [file Presentation_1.pptx]

## Slide 1
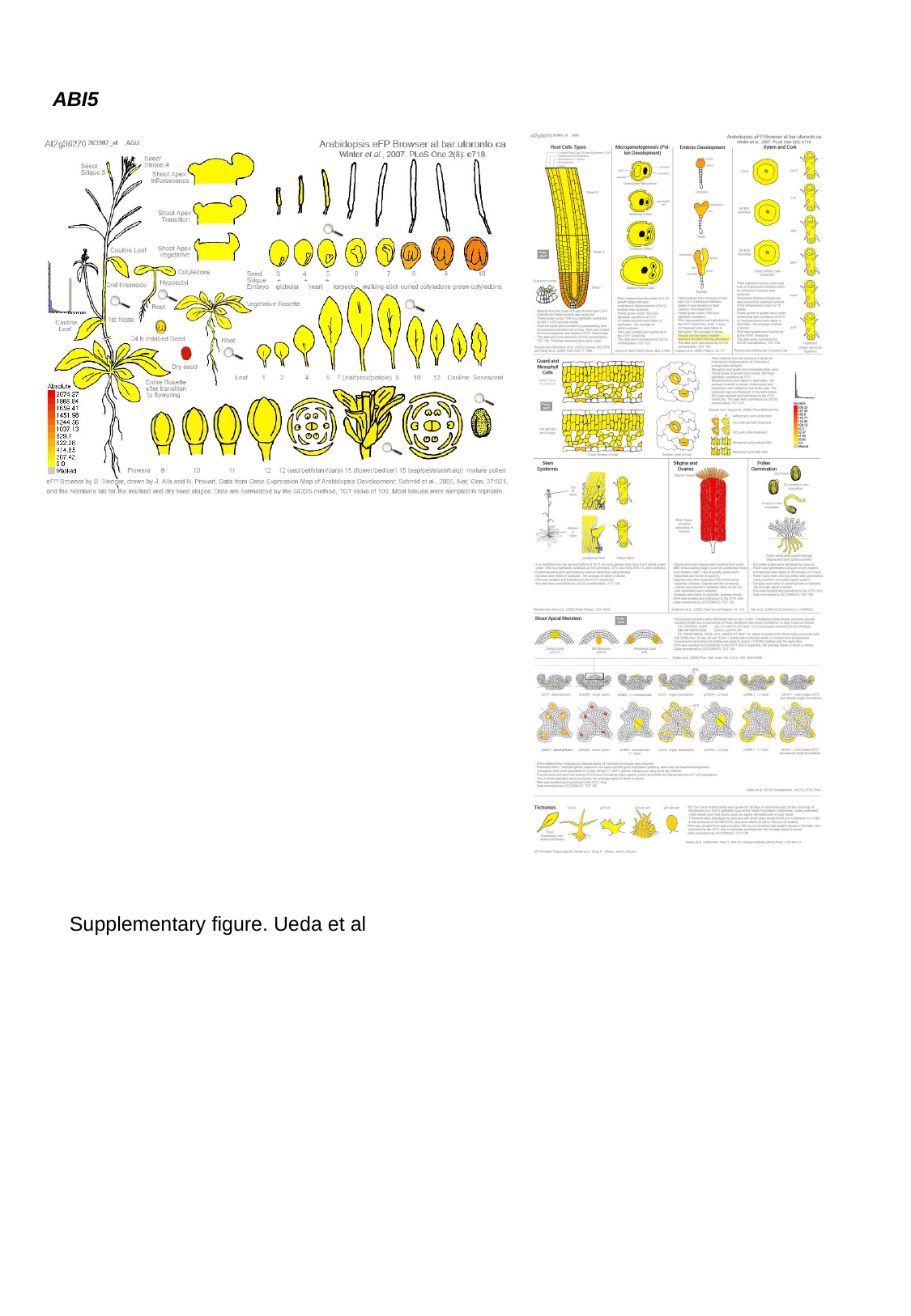

ABI5
Supplementary figure. Ueda et al

## Slide 2
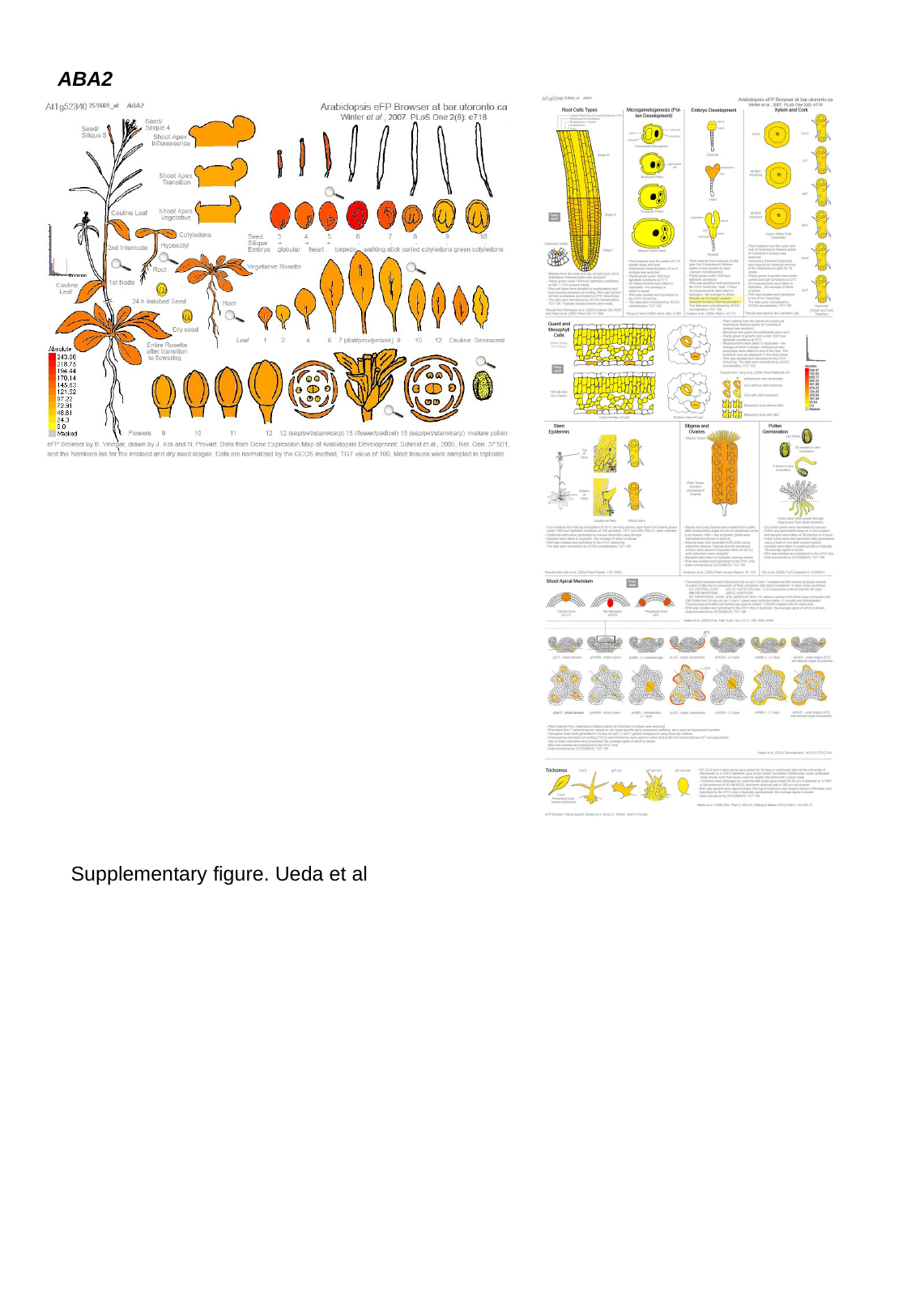

ABA2
Supplementary figure. Ueda et al

## Slide 3
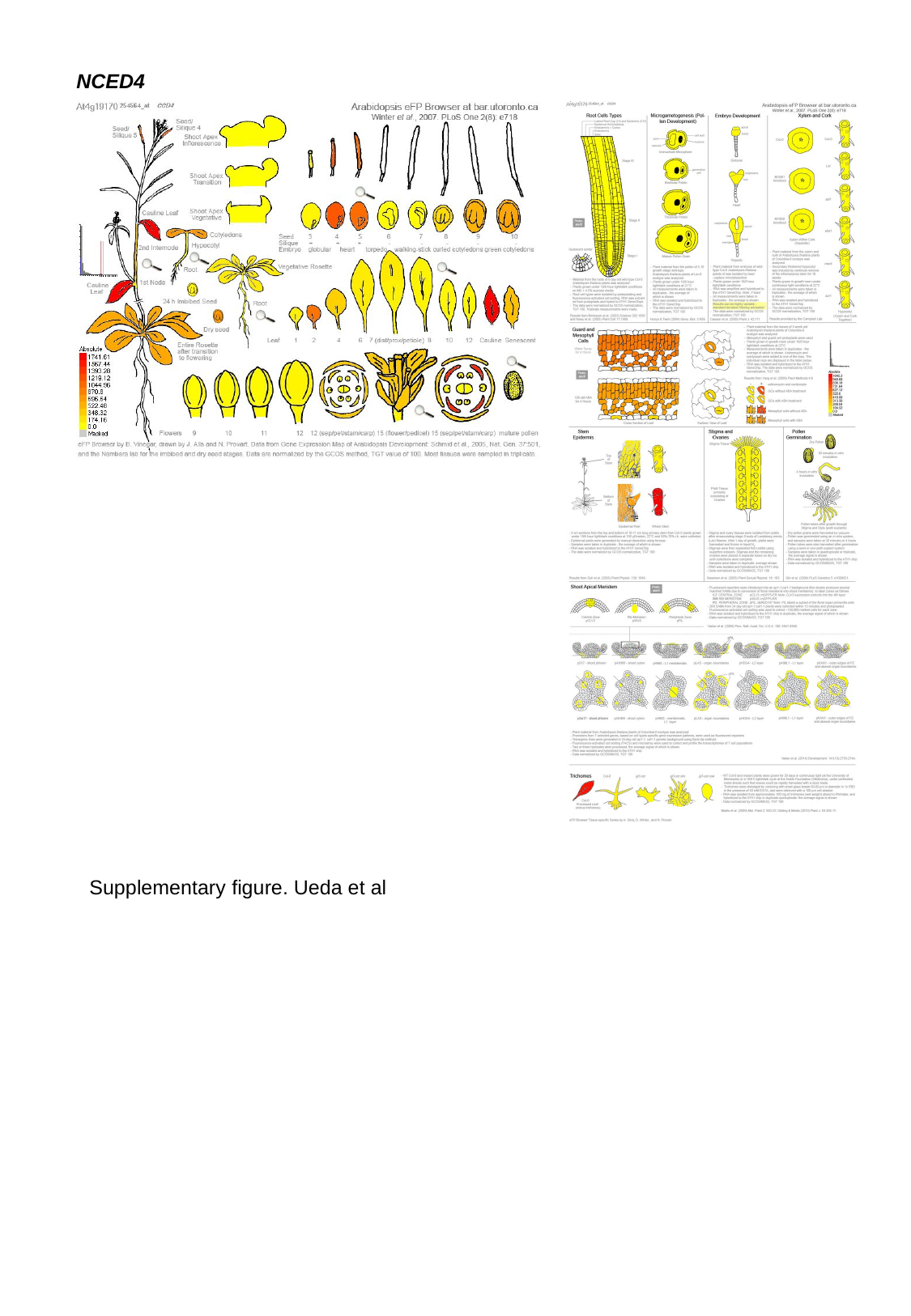

NCED4
Supplementary figure. Ueda et al

## Slide 4
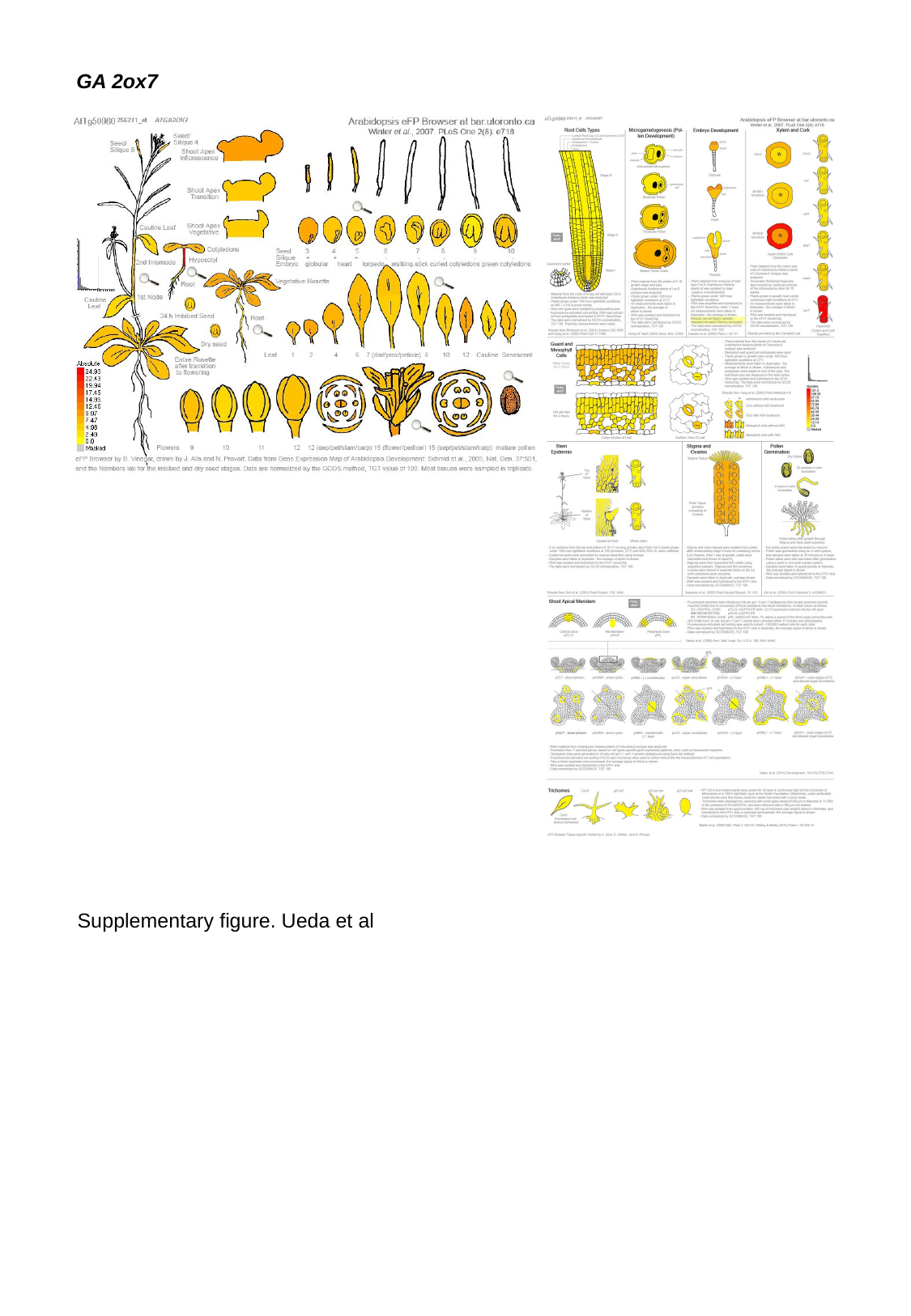

GA 2ox7
Supplementary figure. Ueda et al

## Slide 5
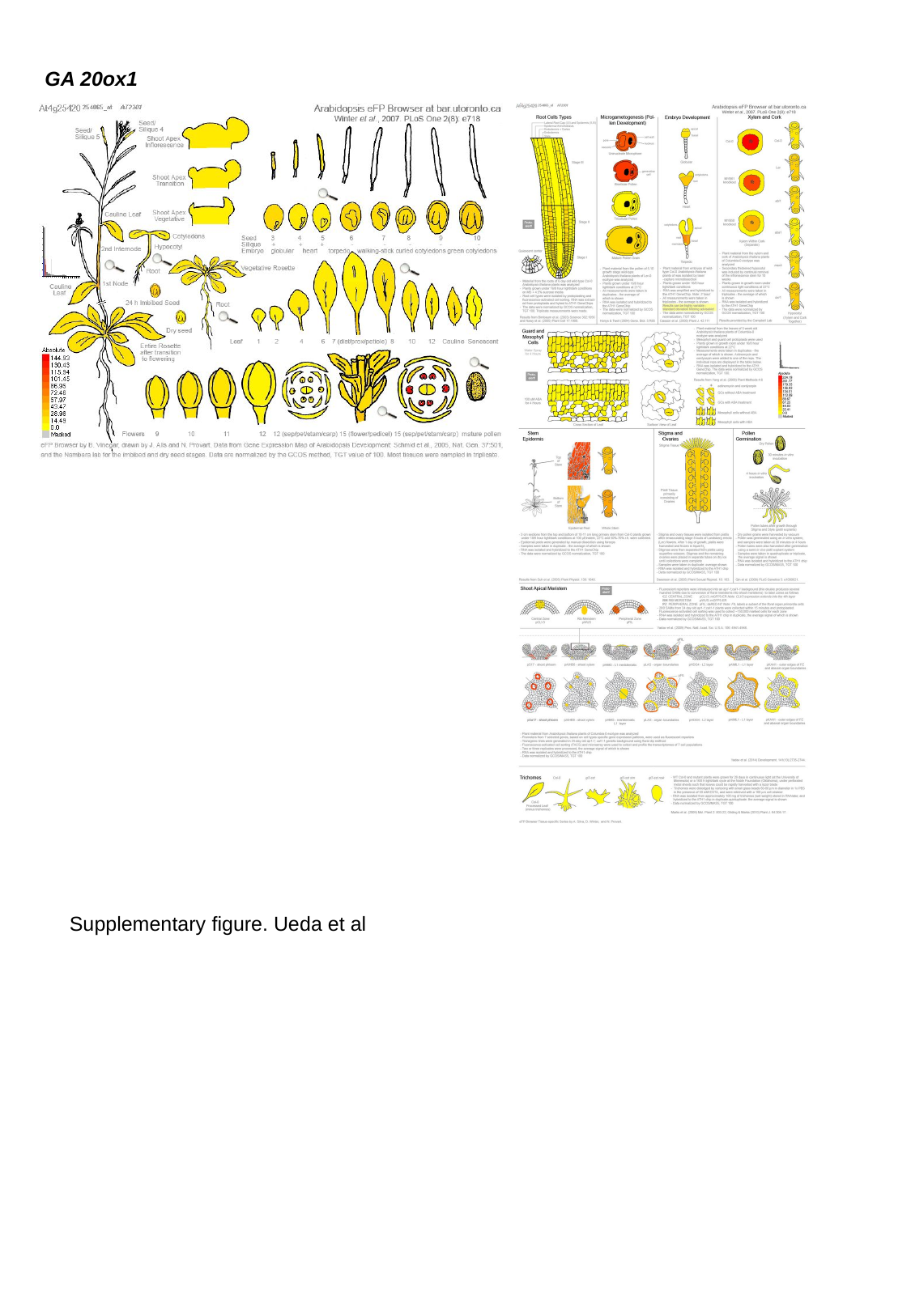

GA 20ox1
Supplementary figure. Ueda et al

## Slide 6
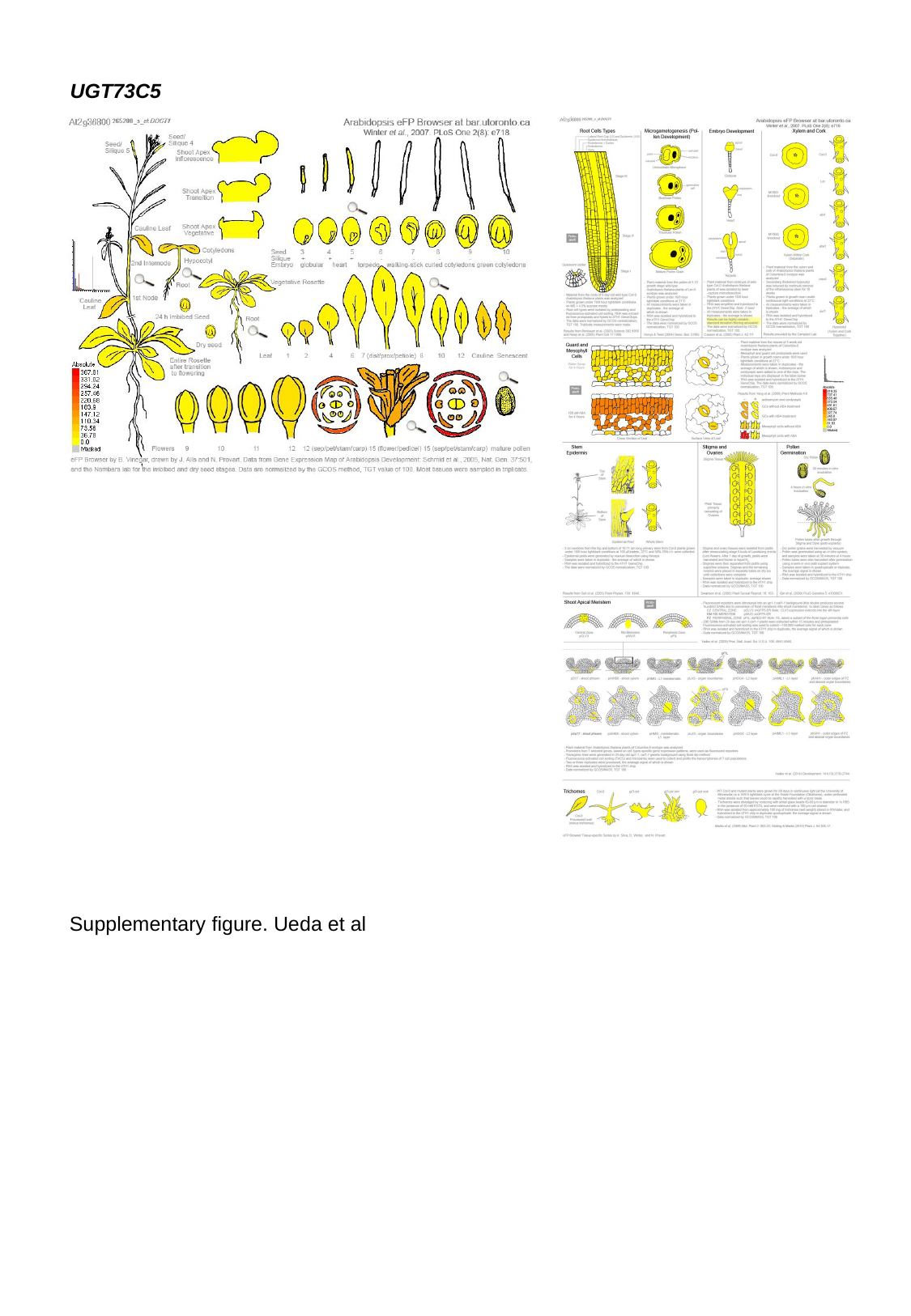

UGT73C5
Supplementary figure. Ueda et al

## Slide 7
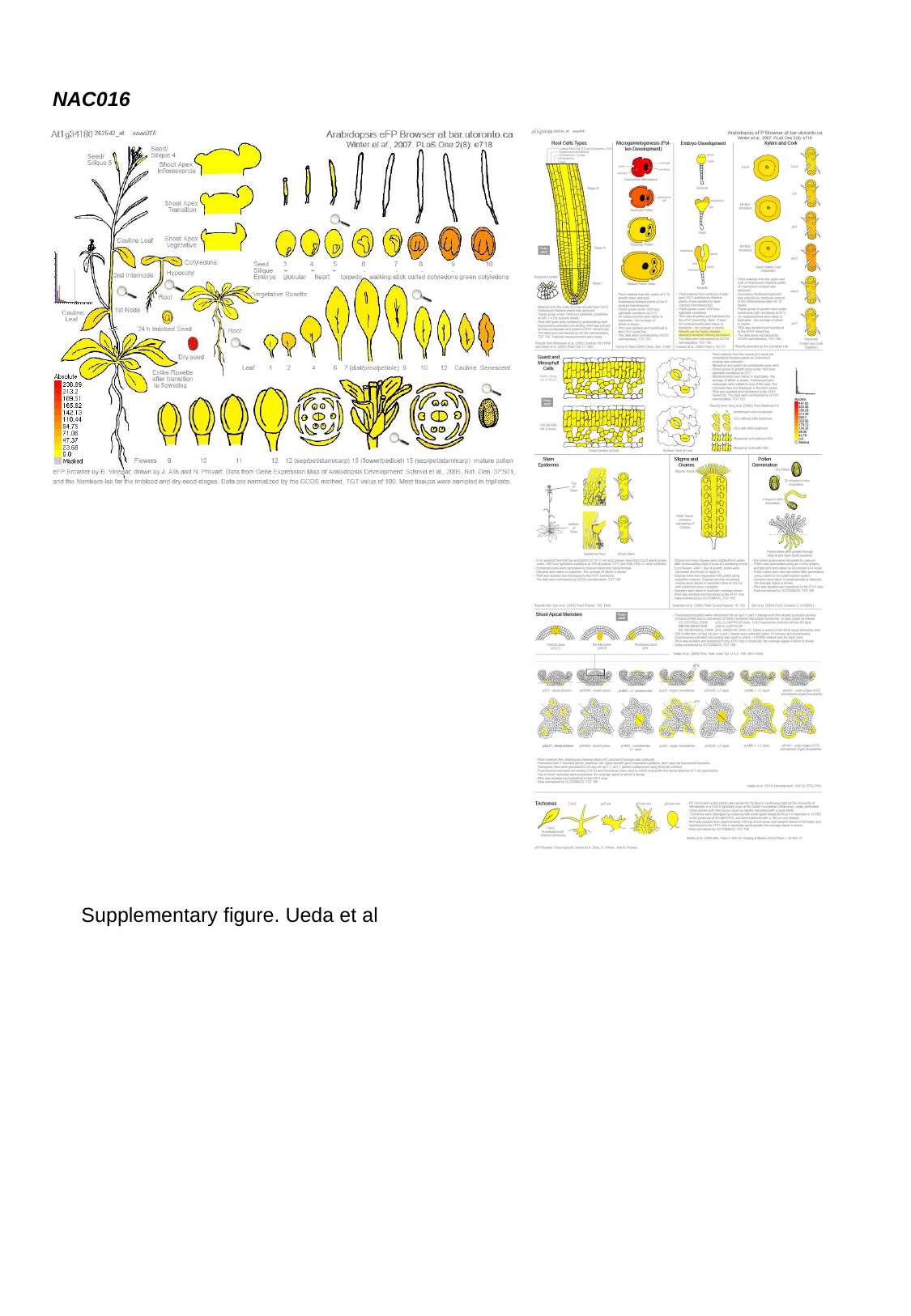

NAC016
Supplementary figure. Ueda et al

## Slide 8
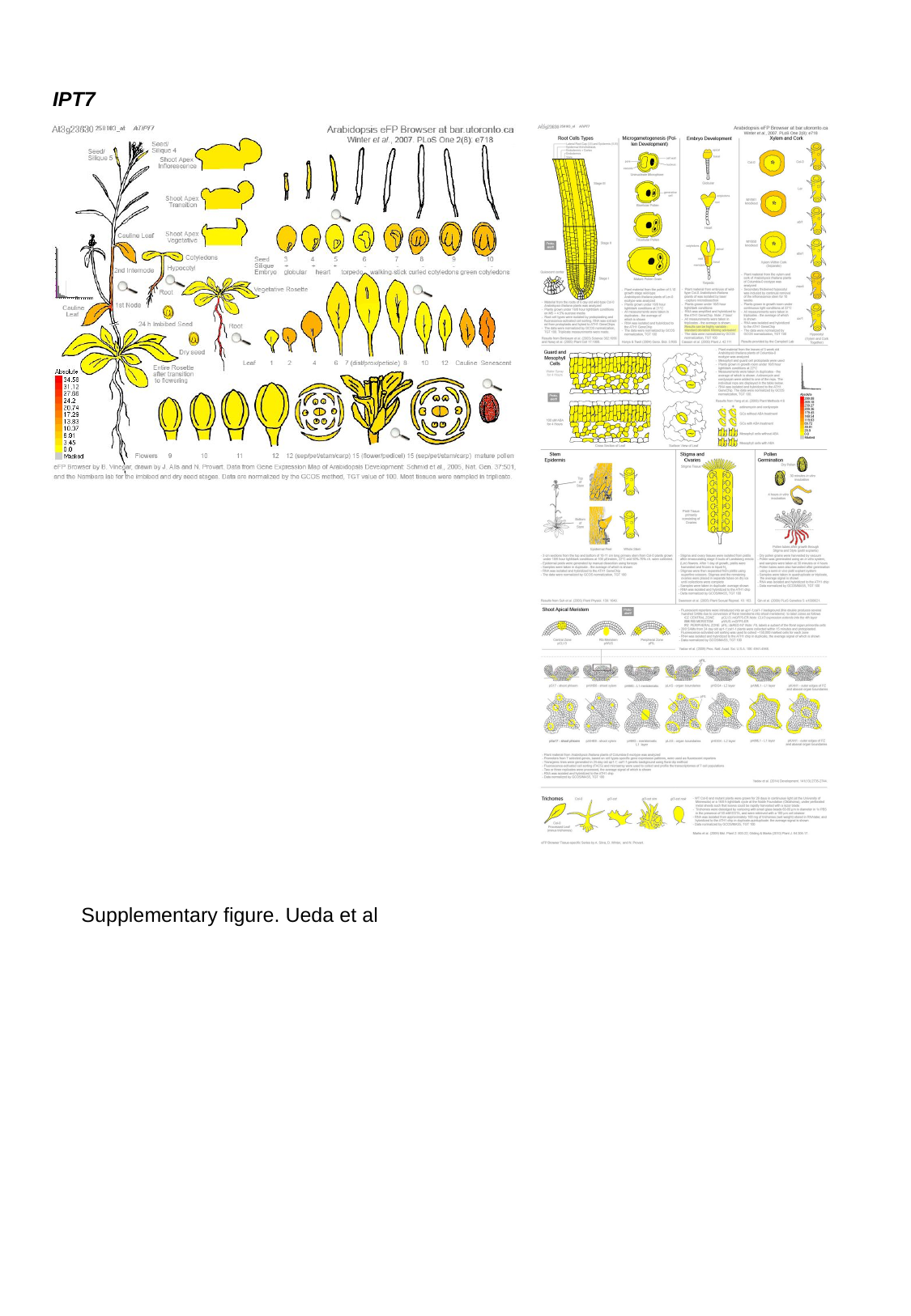

IPT7
Supplementary figure. Ueda et al
